# Harmonisation of Dietary Intake Data in Pregnant Women: Data from the Brazilian Maternal and Child Nutrition Consortium—BMCNC

**DOI:** 10.3390/nu18132068

**Published:** 2026-06-24

**Authors:** Bruna Lazzeri, Helena M. Constante, Monica A. Batalha, Juliana S. Vaz, Caroline B. Gomes, Silmara S. B. S. Mastroeni, Marco F. Mastroeni, Gilberto Kac, Daniela S. Sartorelli, Michele Drehmer, Brazilian Maternal and Child Nutrition Consortium

**Affiliations:** 1 Postgraduate Program in Epidemiology, School of Medicine, Federal University of Rio Grande do Sul, Porto Alegre 90035-003, Rio Grande do Sul, Brazil; bruna.lazz@gmail.com; 2 Department of Sociological Studies, The University of Sheffield, Sheffield S10 2TN, UK; lenaconstante@gmail.com; 3 Department of Epidemiology and Population Health, Albert Einstein College of Medicine, New York, NY 10461, USA; monicaabatalha@gmail.com; 4 Postgraduate Program in Nutrition and Foods, Faculty of Nutrition, Federal University of Pelotas, Pelotas 96010-610, Rio Grande do Sul, Brazil; juliana.vaz@gmail.com; 5 Postgraduate Program in Public Health, Botucatu Medical School, São Paulo State University (UNESP), Botucatu 18618-687, São Paulo, Brazil; carol.bgomes@yahoo.com.br; 6 Nutrition Course, University of Joinville Region (UNIVILLE), Joinville 89219-710, Santa Catarina, Brazil; silmara.mastroeni@gmail.com; 7 Postgraduate Program in Health and Environment, University of Joinville Region (UNIVILLE), Joinville 89219-710, Santa Catarina, Brazil; marco.mastroeni@gmail.com; 8 Nutritional Epidemiology Observatory, Josué de Castro Nutrition Institute, Federal University of Rio de Janeiro, Rio de Janeiro 21941-853, Rio de Janeiro, Brazil; gilbertokac@gmail.com; 9 Department of Social Medicine, Ribeirão Preto Medical School, University of São Paulo, Ribeirão Preto 14040-900, São Paulo, Brazil; daniss@fmrp.usp.br; 10 Postgraduate Studies Program in Food, Nutrition and Health, Department of Nutrition, School of Medicine, Federal University of Rio Grande do Sul, Porto Alegre 90035-003, Rio Grande do Sul, Brazil

**Keywords:** data harmonisation, dietary data, pregnant women, food frequency questionnaire, food consumption screeners, nutritional epidemiology, Brazilian Maternal and Child Nutrition Consortium

## Abstract

**Background/Objectives**: This study describes the process of harmonising data from food consumption screeners (FCSs) and food frequency questionnaires (FFQs) in pregnant women, highlighting challenges and strategies. **Methods**: It is a methodological, descriptive study on the harmonisation of individual food intake data. The data were divided into two datasets: FCS and FFQ. FCS responses were categorised as “never/almost never”, “1–4 days per week”, and “≥5 days per week”. FFQ data were harmonised by deriving variables in grams per day. Outliers were identified using z-scores for total harmonised caloric intake exceeding ±2 standard deviations. The distribution and heterogeneity of the derived variables were assessed using multilevel models. **Results**: Data were drawn from 12 studies conducted in Brazil, part of the Brazilian Maternal and Child Nutrition Consortium (BMCNC). The sample included pregnant women aged 18 years or older, at any stage of pregnancy. The final harmonised datasets comprised eight studies (*n* = 5484) with FCS data and four studies (*n* = 1759) with FFQ data. Most food categories in the FCS dataset had comparable frequencies across studies, with differences observed for natural juices, soft drinks, and sweetened beverages. In the FFQ data, the largest variations in daily consumption were found for leafy vegetables, sweetened beverages, and soft drinks. Heterogeneity ranged from less than 0.01% for beans (FCS) to 15.5% for fruits and natural juices (FFQ). **Conclusions**: By enabling standardised analyses across diverse Brazilian populations, the harmonised BMCNC datasets provide a valuable resource for investigating nutritional inequities and supporting future research to improve maternal and child nutrition.

## 1. Introduction

Maternal diet plays a crucial role in offspring development, influencing birth weight and health outcomes across the life course [1,2,3,4]. However, investigating the associations between dietary factors and health outcomes presents challenges, as these effects tend to be modest and require large and diverse samples to be adequately examined [5].

These challenges are particularly relevant in settings marked by pronounced social and regional inequalities. In Brazil, maternal and child dietary patterns vary substantially according to income, educational attainment, food insecurity, race and ethnicity, and geographic region. Women and children living in socioeconomically disadvantaged households are more likely to experience lower diet quality and poorer nutrition-related health outcomes, reflecting structural barriers to accessing healthy and culturally appropriate foods [6,7].

In this context, the establishment of research consortia and data harmonisation initiatives has emerged as an important strategy to increase analytical power and improve comparability across studies. By integrating data from different populations, harmonisation enables the investigation of dietary patterns across diverse socioeconomic, cultural, and environmental contexts, thereby facilitating the identification of nutrition-related inequities and their determinants [8,9].

Although harmonisation does not alter the quality of the original data, it allows the identification of inconsistencies, enables the application of more advanced analytical methods, and supports comparative analyses across populations [10]. Furthermore, the integration of multiple datasets promotes collaboration among research groups and contributes to the more efficient use of existing data [8,9,11].

Most harmonisation initiatives have been conducted in high-income settings, parti-cularly within international consortia, such as the Global Pregnancy Collaboration (CoLab) and the LifeCycle Project [12,13]. However, similar initiatives remain limited in low- and middle-income countries, where greater socioeconomic and cultural diversity may influence dietary patterns and their associated health outcomes.

In Brazil, the Brazilian Maternal and Child Nutrition Consortium (BMCNC) represents the first initiative to retrospectively harmonise primary dietary intake data in the field of maternal and child health. By integrating information from cohorts conducted in different regions and sociocultural settings, the consortium provides a unique opportunity to investigate dietary intake and nutritional outcomes across populations with varying social and economic characteristics. This approach is particularly relevant for examining how structural determinants shape maternal nutrition and contribute to nutrition-related inequities among pregnant women, mothers, and children. Given the metho-dological complexity of this process, detailed documentation of the decisions adopted is essential to ensure transparency, reproducibility, and usefulness for future research [14,15]. The harmonised datasets developed within the BMCNC provide a structured basis for future multicentre analyses of maternal dietary intake during pregnancy and its associations with maternal and child health outcomes. Potential applications include investigations of fruit and vegetable consumption in relation to birth outcomes, analyses of gestational weight gain according to selected dietary indicators, and the derivation of dietary profiles based on harmonised food groups. In addition, these data may support ana-lyses of how dietary patterns differ according to socioeconomic and regional characteristics, generating evidence to inform nutrition equity–oriented policies, programmes, and community-based interventions aimed at improving maternal and child nutrition in Brazil and reducing nutrition-related disparities across populations.

Thus, this study aimed to describe the method used to harmonise data from food consumption screeners (FCS) and selected items from food frequency questionnaires (FFQ) in pregnant women within the BMCNC, as well as to present the main challenges and strategies involved in this process.

## 2. Materials and Methods

### 2.1. Population and Study Design

This is a methodological harmonisation study using secondary data analysis, conducted within the Brazilian Maternal and Child Nutrition Consortium (BMCNC), a collaborative research network focused on maternal and child nutrition in Brazil [16].

The study aimed to harmonise food consumption data from pregnant women (≥18 years old, at any gestational stage) using datasets from observational studies and intervention studies (control groups only) included in the consortium.

Eligible studies were required to:

- include dietary data collected during pregnancy
- provide access to raw food consumption data
- include sufficient methodological documentation (e.g., questionnaires, variable dictionaries)

A total of 14 studies initially met these criteria. Two studies were excluded: one due to the absence of raw food consumption data (only derived nutrient data available), and another because it assessed only specific fatty acids rather than overall dietary intake [17,18,19,20,21,22,23,24,25,26,27,28].

After dataset integration, individual-level exclusions were applied:

- multiple pregnancies (*n* = 14)
- women aged < 18 years or with missing age (*n* = 728)
- missing data for all FFQ variables (*n* = 4)

### 2.2. Data Processing and Storage

To ensure data protection and uphold the principles of open science and FAIR (Findable, Accessible, Interoperable, and Reusable) [29], the BMCNC established a virtual data repository to receive datasets from all included studies. Dataverse, an open-source web application for data storage and processing (https://dataverse.harvard.edu/), was implemented at the Nutritional Epidemiology Observatory of the Federal University of Rio de Janeiro (UFRJ) in collaboration with researchers from the Research Data Network of the Federal University of Rio Grande do Sul (UFRGS). Each principal investigator (PI) of the participating studies was required to upload their raw dataset, along with a variable dictionary, the questionnaire used, and, where available, a methodological paper describing the study.

### 2.3. Exploration of Food Consumption Data and Variable Definition

To achieve final harmonisation, several steps were undertaken. Firstly, all BMCNC studies containing food consumption data during pregnancy were reviewed and grouped according to the data collection method. Two main groups were identified: studies using FFQ and studies assessing food consumption frequency through short questionnaires, referred to as FCS.

Secondly, the food consumption data collection instruments were identified and assessed in terms of validation status, frequency of administration, sample size, and study period. This information was gathered in collaboration with the PIs of each study.

Thirdly, food groups of interest were selected based on their presence across BMCNC studies, relevance during pregnancy, and role as specific dietary markers. The selection was guided by national [30,31] and international [32,33] dietary guidelines, leading to the inclusion of the following food groups for harmonisation: fruits, natural juices, vegetables, beans, fish, dairy products, and sweetened beverages.

Subsequently, after analysing the available variables and harmonisation options, it was decided to construct two independent databases: one comprising studies that assessed diet using FCS, and another comprising studies using FFQ, as these instruments capture dietary intake differently. One key difference observed was that FCS studies did not include portion size data, preventing the calculation of daily gram intake across all questionnaires. Additionally, FCS studies assessed food groups (e.g., fruits), whereas FFQ studies recorded individual food items (e.g., bananas, apples, oranges) separately. Consequently, the nature of the dietary data collected differed between instruments. Finally, after merging individual datasets, harmonised food consumption variables were created separately for the FCS and FFQ databases, following the procedures described below (Supplementary Figure S1).

### 2.4. Harmonisation

For the data harmonisation process, the following elements were considered:

(i) similarity in question formulation,
(ii) whether responses included only consumption frequencies or also portion sizes,
(iii) diversity in response categories, and
(iv) the feasibility of creating derived variables, such as calculating grams per day based on portion sizes.

Below, detailed information is provided on the harmonisation process for food consumption screeners (A) and food frequency questionnaires (B).

A. Food consumption screeners—FCS

The first aspect analysed was the formulation of the questions in the questionnaires. Most studies used the question: “How many days per week did you consume (…)?” However, one questionnaire asked: “How often do you consume each of the foods below?”, while another only provided a table with response options. Although these formulations differed, some questionnaires that inquired about days per week also offered response options in terms of times per week. After discussions with the PIs, it was decided that, despite the differences in phrasing, the underlying objective was the same: to capture weekly consumption frequency in days. Based on this, these formulations were considered equivalent and were standardised during the harmonisation process to ensure comparability between studies.

The second element analysed was the response options. In all studies, participants were presented with five response options for each screener. Although these options referred to weekly consumption frequency, the specific categories varied across studies. To harmonise the responses, they were reorganised into three standard categories: “never/almost never”, “1–4 days per week”, and “≥5 days per week” (Supplementary Material—Box S1).

The third element involved assessing the food groups included in each consumption screener, considering the different question formats across studies. For example, when addressing the “fruits” variable, studies that grouped fruits and juices in the same question were excluded. For these cases, a “fruits and natural juices” variable was created. In studies where fruits and juices were assessed separately, the highest reported consumption frequency was used for the combined variable. For example, if a participant reported “never/almost never” for natural juice but “1–4 days per week” for fruit consumption, the combined variable was assigned “1–4 days per week”. A similar approach was applied to the “soft drinks” and “sweetened beverages” variables, where the latter included grouped variables such as “artificial juices and soft drinks” or the most frequent response when assessed separately. Regarding vegetables, only one variable—“all vegetables”—was created, as only the study by Gomes et al. (2021) [18] distinguished leafy vegetables separately.

Initially, 13 variables were established to represent different food groups or specific foods. A minimum threshold of three studies was adopted to ensure that each harmonised variable was informed by more than one independent data source while allowing for the assessment of between-study variability. Including variables present in fewer studies would increase the influence of study-specific definitions and limit the interpretability of heterogeneity estimates. Additionally, the grouped “dairy products” variable was excluded because, in studies where dairy foods were assessed either separately or as a single category, milk consistently emerged as the most consumed item.

At the end of the process, eight variables were included in the harmonised dataset: (1) fruits; (2) natural juices; (3) fruits and natural juices (grouped); (4) vegetables; (5) beans; (6) milk; (7) soft drinks; and (8) sweetened beverages.

B. Food frequency questionnaire—FFQ

All four studies that employed an FFQ showed variations in the number of items listed, frequency categories, and portion sizes (Supplementary Material—Boxes S2 and S3). As in the FCS dataset, foods or food groups reported in fewer than three studies, such as seafood and artificial juices, were excluded. Additionally, foods with very low average intake (<10 g/day) were excluded to minimise the influence of sporadic consumption and reduce the impact of measurement error inherent to FFQ data. At such low levels of intake, estimates are more susceptible to random variation and misreporting, which may compromise comparability across studies and lead to overinterpretation of nutritionally negligible contributions. At the end of the process, eleven variables were created to represent the food groups initially selected by the BMCNC: (1) fruits; (2) natural juices; (3) fruits and natural juices (grouped); (4) leafy vegetables; (5) all vegetables; (6) soft drinks; (7) sweetened beverages; (8) beans; (9) fish; (10) milk; and (11) dairy products.

The units of measurement for the FFQ-derived food consumption variables were expressed in grams per day. Initially, for each food item, consumption in grams was calculated using the following formula: Grams = quantity × weight × frequency. The values obtained from this formula were then converted into grams per day by adjusting for different consumption frequency intervals (e.g., twice per week, five times per week, twice per month). This conversion was performed by dividing the total amount consumed (in grams) by the corresponding number of days for each frequency interval.

Next, food items were grouped by type (e.g., fruits: bananas, apples), and the total consumption in grams for each food group was calculated by summing the individual items within that group. Total energy intake (kcal) was calculated by converting the weight (grams) of each food item into kilocalories using the Brazilian Food Composition Table (TBCA) (http://www.tbca.net.br/). The following formula was applied: Energy from food item = (grams of food × kcal in 100 g of food)/100. For instance, in the case of avocado: Energy from avocado = (grams of avocado × kcal in 100 g of avocado)/100.

The total caloric intake for each pregnant woman was calculated as the sum of all food items consumed. Lastly, for studies that administered more than one FFQ during pregnancy, the adopted criterion was to use the last measurement collected, thus avoiding repeated measures.

### 2.5. Statistical Analysis

The FCS variables were assessed according to three categories: “never or almost never”, “1 to 4 days per week”, and “≥5 days per week”, as well as their relative frequency in the final dataset.

For the FFQ data, an outlier analysis was conducted based on total caloric intake. A value was considered an outlier when the variable presented values <−2 or ≥+2 standard deviations (SD), resulting in 21 observations with values above 9224 kcal. No values were identified below −2 SD. Additionally, means and standard deviations were calculated for the derived food consumption variables, measured in each dataset and in the final combined dataset.

In the analysis of heterogeneity for the FCS database, a multilevel logistic regression model was fitted, including the study identifier as a random effect. The variance partition coefficient (VPC) was calculated to assess the proportion of total variation explained by differences between studies. In the case of the FFQ database, a mixed-effects model was employed, with the dependent variable being grams of food and the study identifier included as a random effect. The intraclass correlation coefficient (ICC) was calculated to quantify the proportion of total variance attributable to differences between studies. The VPC and ICC estimates were interpreted descriptively to characterise the proportion of variability attributable to differences between studies. As no universally accepted cut-offs are available for interpreting between-study heterogeneity in harmonised dietary datasets, values around 10% were used solely as a pragmatic reference to facilitate the interpretation of between-study variability. These values were not used as formal criteria for harmonisation validity, study inclusion, or analytical decision-making. Statistical analyses were conducted using Stata version 15.0.

### 2.6. Ethical Approval

This study was conducted in accordance with the principles laid down in the Declaration of Helsinki. The analysis of secondary data was approved by the Research Ethics Committee of the Maternity Teaching Hospital at the Federal University of Rio de Janeiro (protocol number: 33897420.4.0000.5275). Each study incorporated into the BMCNC received individual approval from its respective institutional research ethics committee. Formal agreements were signed between each study’s principal investigator and the BMCNC for data sharing. All analyses were conducted using de-identified data in a secure environment with restricted access.

## 3. Results

**Figure 1:**
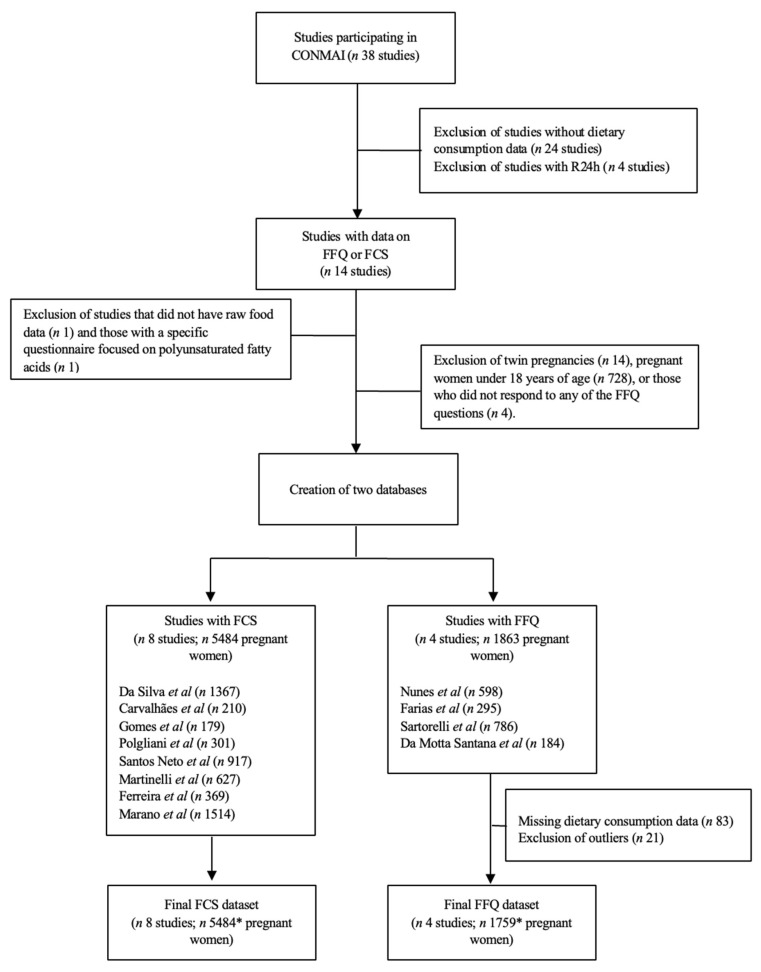
Flowchart of the data cleaning steps for the combined dataset. BMCNC: Brazilian Maternal and Child Nutrition Consortium; FFQ: Food Frequency Questionnaire; FCS: Food Consumption Screeners. * The number of participants (*n*) may vary depending on the availability of the dietary intake variable.

A total of twelve studies were included, encompassing 7243 pregnant women, with 5484 from FCS data (8 studies) and 1759 from FFQ data (4 studies) (Figure 1). The studies were conducted between 2006 and 2014, representing three regions of the country (Table 1).

In the FCS dataset, most food groups showed a clear pattern of frequent consumption, with the highest proportions observed in the “≥5 days per week” category (Table 2).

Overall, the VPC estimates suggested relatively limited between-study variability across the harmonised FCS variables (Table 3).

Fruits, vegetables, and beans were among the most frequently consumed food groups, with a large proportion of women reporting consumption ≥ 5 days per week. Beans showed minimal between-study variability. Milk consumption also tended to be frequent, although with slightly greater variation across studies.

In contrast, greater variability was observed for beverage consumption. Natural juices, sweetened beverages, and soft drinks showed greater between-study variability, with a higher proportion of participants reporting intermediate consumption frequencies (1–4 days per week). Soft drinks, in particular, were less frequently consumed on a daily basis compared to other food groups.

In the FFQ dataset, greater variability in intake was observed across studies, both in terms of mean consumption and dispersion (Table 4). This was reflected in greater between-study variability for some variables, particularly fruits and combined “fruits and natural juices”, possibly related to differences in dietary assessment methods and food item composition across studies (Table 3).

Leafy vegetables and sweetened beverages showed moderate variability, while fish consumption was relatively consistent across studies, with low heterogeneity. Overall, the FFQ-based estimates revealed greater between-study variation compared with the FCS dataset, likely reflecting differences in measurement methods and level of dietary detail.

## 4. Discussion

This study presents a harmonised dietary dataset of pregnant women from multiple regions of Brazil. The results suggest that, despite methodological differences among the included studies, it was possible to derive harmonised dietary indicators with relatively comparable distributions across studies.

This advancement is particularly relevant in a context characterised by wide regional, socioeconomic, and cultural diversity, where persistent disparities in food access, diet quality, and nutrition-related health outcomes affect pregnant women and children. In this setting, the lack of integrated databases has limited the capacity to investigate nutritional inequities and their structural determinants across populations.

Consistent patterns were observed for key food groups, such as fruits, vegetables, and legumes, suggesting that the harmonisation approach was able to preserve central characteristics of the Brazilian dietary pattern across different studies. These results were also comparable to data from the Food and Nutrition Surveillance System (SISVAN) in Brazil, referring to dietary intake among 35,543 pregnant women followed in primary health care in 2020 [34]. These findings support the plausibility of the harmonised dietary indicators and suggest that the observed distributions are broadly compatible with national dietary data.

In contrast, greater variability was identified in estimates derived from FFQ, reflec-ting differences in questionnaire structure, number of items, and portion size estimation. These differences may be partially attributed to variability in the number of items included, highlighting the importance of standardised research instruments. The inclusion of an excessive number of fruit varieties in some studies tends to overestimate total fruit consumption, as the estimated quantity is approximately proportional to the number of questions asked [35]. A potential approach to mitigate this heterogeneity in future studies would be to standardise the number of items across studies, prioritising those most frequently consumed, as suggested by Thompson et al. (2017) [35].

Regarding beverage consumption, such as natural juices, sweetened beverages, and soft drinks, greater discrepancies were observed in frequency distributions across regions, with fruit juice consumption being more prominent in the Northeast and sweetened be-verages more common in the Southeast and South. These variations are not only sources of heterogeneity between datasets but also reflect regional particularities. Harmonised datasets from several European countries have similarly demonstrated clear geographical variability [36,37]. For example, in the study by Mertens et al. (2021), there was substantial variation between countries in average daily consumption of foods such as fruits (118–199 g/day), vegetables (95–239 g/day), fish (12–45 g/day), dairy products (129–302 g/day), and sweetened beverages (48–224 mL/day) [36].

Soft drink consumption, when analysed individually, may have limited utility despite showing low heterogeneity in the multilevel analysis. This variable was assessed in only three of the eight studies included in the FCS dataset. Of these, two were conducted in the same city, while the third showed a different distribution across consumption categories compared with the others. In the FFQ dataset, soft drink consumption was not collected in all studies, and the intake reported by Santana et al. (2015) [28] was considerably lower than in the other studies.

The limited number of studies assessing soft drink consumption is particularly concerning, as it may compromise the representativeness and comparability of this indicator. This limitation is especially relevant given that the most recent national report on the nutritional status of pregnant women in Primary Health Care in Brazil showed that more than half (56%) reported consuming sugar-sweetened beverages, including soft drinks, on the day prior to the interview, and 76% reported consuming ultra-processed foods in general, both markers of unhealthy dietary patterns [34]. These findings underscore the importance of generating harmonised dietary indicators that enable the investigation of socioeconomic and regional disparities in the consumption of ultra-processed foods, which are increasingly recognised as contributors to nutritional inequities and adverse maternal and child health outcomes.

Beans showed minimal between-study variability in the harmonised FCS dataset. This consistency is due to the uniform distribution of values across categories, with minimal between-study variability according to the descriptive heterogeneity estimates. One factor likely contributing to this high similarity is the cultural role of beans in Brazil, which, together with rice, forms the country’s typical meal. Despite a declining trend in recent years among the adult population [37], beans remain widely consumed, especially among pregnant women. Data from pregnant women followed in primary health care revealed that 80% consumed beans on the day prior to the consultation [32]. These findings are consistent with the harmonised data, which indicate a consumption rate of 84.4% on five or more days per week. It is also worth noting that national dietary guidelines for pregnant women recommend encouraging daily consumption of beans or other legumes, preferably at lunch and dinner [38].

In the FFQ dataset, fish showed minimal between-study variability. However, unlike beans, which showed high consumption frequency across all FCS studies, the average daily fish intake was low (mean of 17 g/day). Therefore, the use of the fish variable should be approached with caution in future studies using BMCNC data. A weekly assessment of fish consumption is suggested, allowing comparisons with recommendations advoca-ting the intake of at least one portion of omega-3-rich fish per week, such as those from the Institute of Medicine (2020) [39]. In the FCS dataset, this variable was not harmonised, as it was present in only two studies.

Numerous challenges arise in the construction of a unified and methodologically robust dataset. A 2022 review on dietary data harmonisation methods by Gurugubelli et al. [8] described challenges similar to those encountered in the present study. These range from practical and logistical issues, such as coordinating multiple researchers, scheduling recurring meetings, ensuring funding, data security, and management concerns, to challenges directly related to data handling and interpretation. The latter includes the use of different research instruments, limitations in performing certain analyses, and constraints in applying new statistical models due to the inability to standardise all necessary variables.

An example of the impact of instrument diversity on data harmonisation was the second consumption category in the FCS database (“1 to 4 days per week”), which resulted in a loss of information on intermediate consumption levels, as these could not be fully captured due to variability in the intervals used across studies. Even in comprehensive instruments such as FFQ, the lack of uniformity in data collection posed methodolo-gical challenges due to differences in the number of questions, frequency categories, and portion sizes. Similar issues have been reported in studies such as ALPHABET [37], in which FFQ ranged from 43 to 293 food items, with 5 to 9 response categories, and portion sizes varying by country or region, leading to discrepancies in estimated intake.

Another study describing similar challenges is that of Dofkova et al. (2016) [40], which aimed to propose a methodology for establishing harmonised national food lists and to test the feasibility of the proposed procedures in five European countries with different consumption patterns. From the outset, it was recognised that the format of available dietary datasets differed between countries. A diverse proportion of composite foods (traditional dishes) across countries was identified as a limitation. Similarly, Olsen et al. (2014) [41], who compared two Nordic dietary databases (Denmark and Norway), found that basic harmonisation resulted in 63 distinct food groups, later aggregated into 31 broader groups to maximise comparability between cohorts.

Although the challenges of data harmonisation are substantial, its benefits must also be acknowledged. The BMCNC was initially established to harmonise gestational weight gain data and develop reference curves applicable to the Brazilian context, representing an important advance over the use of standards derived from other populations [42].

In the field of dietary data, the advantages of harmonised datasets have been widely demonstrated in high-income settings, particularly in European initiatives [14,37,40,41,43,44], where such approaches are more established. However, evidence from low- and middle-income countries remains limited. In this context, the present study contributes to expanding the availability of harmonised dietary data in underrepresented populations and provides a valuable infrastructure for examining how social and structural determinants influence maternal diet and nutrition-related health inequalities among pregnant women, mothers, and children included in the BMCNC.

Beyond analytical advantages, harmonisation efforts also promote collaboration among research groups, enable data reuse, and support the implementation of FAIR principles [29], aligning with current global trends in data-driven research.

At the same time, important limitations must be acknowledged. Potential sources of harmonisation-related bias include differences in questionnaire structure, variation in the number of food items across FFQ, collapsing of response categories in the FCS datasets, and grouping of related food items during variable derivation. These decisions may have reduced the granularity of some dietary measures and may have introduced non-diffe-rential misclassification across studies.

The potential impact of these biases is likely greater for estimates of absolute intake than for relative comparisons between exposure categories, reinforcing the importance of using harmonised indicators primarily for comparative analyses rather than precise quantification. To minimise these effects, harmonisation procedures were guided by conceptual similarity between variables, standardised coding decisions, and a detailed review of the original dietary instruments in collaboration with the principal investigators of each study.

Despite these challenges, this study represents one of the first efforts to harmonise dietary data among pregnant women in a large, socioeconomically diverse middle-income country. By improving comparability across heterogeneous datasets, this database creates new opportunities to investigate dietary intake profiles, nutritional inequities across socioeconomic and regional groups, and their associations with health outcomes among pregnant women, mothers, and children in Brazil.

Beyond its application in a middle-income setting, this study also contributes metho-dologically to the field of dietary data harmonisation. The harmonisation process required the retrospective integration of heterogeneous dietary assessment instruments, including both food consumption screeners and FFQ, each demanding distinct harmonisation stra-tegies. Additional methodological challenges included the reconciliation of grouped versus separately collected food variables, differences in questionnaire structure, and variation in portion size assessment across studies. Furthermore, the use of multilevel variabi-lity estimates provided a structured descriptive approach for characterising residual between-study variability after harmonisation. These challenges are common in multicentre nutritional epidemiology but remain insufficiently described in harmonisation studies conducted in low- and middle-income settings. Future research should focus on developing more standardised dietary assessment instruments, reducing methodological variability, and establishing harmonised data collection protocols in pregnant populations. These advances may strengthen the evidence base generated from harmonised dietary data needed to design, implement, and evaluate equity-oriented policies and community-based interventions aimed at improving maternal and child nutrition and reducing nutrition-related disparities across populations.

## 5. Conclusions

To our knowledge, the BMCNC represents the first maternal and child nutrition consortium in Latin America to harmonise dietary data from pregnant women, resulting in the first unified dietary dataset in Brazil. In a country characterised by vast geographical extent and substantial sociocultural diversity, comparable in scale to continental contexts such as Europe, this initiative addresses a critical gap, given that most existing studies in pregnant women are local and limited to specific regions.

Although the included data were not collected recently, the aim of this study was not to estimate current dietary prevalence but rather to enable future analyses of associations between dietary intake and health outcomes. In this context, the harmonised dataset remains a valuable resource, with patterns comparable to national and international data, reinforcing its relevance for future investigations of dietary intake, maternal and child health, and nutrition-related inequities.

The study also highlights the importance of interinstitutional collaboration and reinforces the need for more standardised dietary assessment tools to improve comparability in future research and strengthen evidence on nutrition-related disparities across populations.

As a methodological study, this work achieved its objectives by providing two harmonised dietary datasets (FCS and FFQ), offering a structured basis for future analyses of dietary intake, nutrition-related inequities, and maternal and child health outcomes. Furthermore, the initiative aligns with FAIR principles, supporting data reuse and contributing to more efficient and collaborative research. By enabling analyses across populations with diverse socioeconomic and regional characteristics, the BMCNC may generate evidence to support nutrition equity–oriented research, policies, and interventions for pregnant women, mothers, and children in Brazil.

## Figures and Tables

**Table 1:** Description of the studies that comprise the dietary intake databases of the Brazilian Maternal and Child Nutrition Consortium (BMCNC).

Study	Year of Data Collection	Region/State/City	Sample	Number of Questions/ Items Collected (Used)
Food Consumption Screeners				
Carvalhaes et al. [17]	2009/10	Southeast/São Paulo/Botucatu	210	18 (4)
Gomes et al. [18]	2013/14	Southeast/São Paulo/Botucatu	179	29 (8) *
da Silva et al. [19]	2010/11	Northeast/Maranhão/São Luís	1367	15 (1)
Ferreira et al. [20]	2013/14	Northeast/Alagoas/Maceió	369	26 (6)
Marano et al. [21]	2007/08	Southeast/Rio de Janeiro/Petrópolis e Queimados	1514	4 (3)
Santos Neto et al. [22]	2010	Southeast/Espírito Santo/Região metropolitana de Vitória	917	13 (6)
Polgliani et al. [24]	2010/11	Southeast/Espírito Santo/Vitória	301	13 (6)
Martinelli et al. [23]	2012/13	Southeast/Espírito Santo/São Mateus	627	13 (6)
Food Frequency Questionnaire				
Nunes et al. [25]	2006/07	South/Rio Grande do Sul/Porto Alegre e Bento Gonçalves	598	88 (41)
Farias et al. [26]	2010/11/12	Southeast/Rio de Janeiro/Rio de Janeiro	295	82 (29)
Sartorelli et al. [27]	2010/11/12	Southeast/São Paulo/Ribeirão Preto	786	95 (35)
Santana et al. [28]	2012/13/14	Northeast/Bahia/Santo Antônio de Jesus	184	78 (32)

* Third Trimester Pregnancy Questionnaire.

**Table 2:** Distribution of dietary intake variables investigated in each dataset according to the final applied categorization, as well as their relative frequency in the final dataset. Food consumption screeners (FCS). Brazilian Maternal and Child Nutrition Consortium (BMCNC), 2026.

	Carvalhaes et al. [17]		Gomes et al. [18]		da Silva et al. [19]		Ferreira et al. [20]		Marano et al. [21]		Santos Neto et al. [22]		Martinelli et al. [23]		Polgliani et al. [24]		Harmonised Data	
	*n*	%	*n*	%	*n*	%	*n*	%	*n*	%	*n*	%	*n*	%	*n*	%	*n*	%
Fruits																		
never or almost never	21	10.00	17	12.78	*.a	.a	26	8.90	.a	.a	90	9.83	75	11.96	18	5.98	247	9.96
1–4 days per week	62	29.52	57	42.86	.a	.a	74	25.34	.a	.a	362	39.52	275	43.86	104	34.55	934	37.68
5 days or more per week	127	60.48	59	44.36	.a	.a	192	65.75	.a	.a	464	50.66	277	44.18	179	59.47	1298	52.36
Natural juices																		
never or almost never	.a	.a	46	34.59	.a	.a	39	13.36	.a	.a	180	19.69	168	26.79	44	14.67	477	21.05
1–4 days per week	.a	.a	58	43.61	.a	.a	62	21.23	.a	.a	365	39.93	226	36.04	119	39.67	830	36.63
5 days or more per week	.a	.a	29	21.80	.a	.a	191	65.41	.a	.a	369	40.37	233	37.16	137	45.67	959	42.32
Fruits and Natural juices																		
never or almost never	21	10.00	12	9.02	.a	.a	9	3.08	217	14.33	46	5.02	52	8.29	9	2.99	366	9.17
1–4 days per week	62	29.52	52	39.10	.a	.a	53	18.15	544	35.93	305	33.30	236	37.64	72	23.92	1324	33.16
5 days or more per week	127	60.48	69	51.88	.a	.a	230	78.77	753	49.74	565	61.68	339	54.07	220	73.09	2303	57.68
Vegetables																		
never or almost never	11	5.24	4	3.01	.a	.a	46	15.75	176	11.63	32	3.50	20	3.19	8	2.66	297	7.44
1–4 days per week	51	24.29	67	50.38	.a	.a	71	24.32	541	35.76	255	27.87	245	39.07	76	25.25	1306	32.72
5 days or more per week	148	70.48	62	46.62	.a	.a	175	59.93	796	52.61	628	68.63	362	57.74	217	72.09	2388	59.83
Sweetened beverages																		
never or almost never	58	27.62	12	9.02	.a	.a	142	48.63	.a	.a	234	25.60	184	29.35	79	26.25	709	28.62
1–4 days per week	116	55.24	61	45.86	.a	.a	104	35.62	.a	.a	423	46.28	240	38.28	131	43.52	1075	43.40
5 days or more per week	36	17.14	60	45.11	.a	.a	46	15.75	.a	.a	257	28.12	203	32.38	91	30.23	693	27.98
Soft drinks																		
never or almost never	58	27.62	32	24.06	.a	.a	142	48.63	.a	.a	.a	.a	.a	.a	.a	.a	232	36.54
1–4 days per week	116	55.24	78	58.65	.a	.a	104	35.62	.a	.a	.a	.a	.a	.a	.a	.a	298	46.93
5 days or more per week	36	17.14	23	17.29	.a	.a	46	15.75	.a	.a	.a	.a	.a	.a	.a	.a	105	16.54
Beans																		
never or almost never	.a	.a	4	3.01	.a	.a	18	6.16	.a	.a	31	3.39	12	1.91	13	4.32	78	3.44
1–4 days per week	.a	.a	24	18.05	.a	.a	37	12.67	.a	.a	102	11.16	86	13.72	26	8.64	275	12.13
5 days or more per week	.a	.a	105	78.95	.a	.a	237	81.16	.a	.a	781	85.45	529	84.37	262	87.04	1914	84.43
Milk																		
never or almost never	30	14.29	14	10.53	138	10.10	74	25.34	.a	.a	163	17.79	90	14.35	61	20.27	570	14.82
1–4 days per week	27	12.86	15	11.28	148	10.83	68	23.29	.a	.a	189	20.63	170	27.11	50	16.61	667	17.35
5 days or more per week	153	72.86	104	78.20	1080	79.06	150	51.37	.a	.a	564	61.57	367	58.53	190	63.12	2608	67.83
Total		100.00		100.00		100.00		100.00		100.00		100.00		100.00		100.00		100.00

*.a: variable not collected.

**Table 3:** Proportion of total variation explained by the difference between studies for each dietary intake variable in the food consumption screeners (FCS) and food frequency questionnaires (FFQ) databases.

Dietary Intake Variable	Heterogeneity (%)	
	FCS	FFQ
Fruits	2.5	13.9
Natural juices	8.1	4.9
Fruits and Natural juices	5.7	15.5
All vegetables	3.4	6.8
Leafy vegetables	*.a	9.4
Sweetened beverages	6.7	8.3
Soft drinks	3.0	2.2
Beans	<0.01	6.5
Fish	.a	0.1
Dairy products	.a	6.0
Milk	5.0	4.5

*.a: variable not collected.

**Table 4:** Distribution of dietary intake variables (in grams/millilitres) in each dataset. Food Frequency Questionnaires· Brazilian Maternal and Child Nutrition Consortium (BMCNC), 2026.

	Nunes et al. [25]					Farias et al. [26]					Sartorelli et al. [27]					Santana et al. [28]					Harmonised Data				
	Mean	SD	* Q1	Median	† Q3	Mean	SD	Q1	Median	Q3	Mean	SD	Q1	Median	Q3	Mean	SD	Q1	Median	Q3	Mean	SD	Q1	Median	Q3
Fruits	735	657	282	528	1000	300	211	139	257	405	371	364	135	274	495	752	684	377	577	920	521	539	180	356	666
Natural juices	256	397	0	103	310	129	165	11	71	165	171	236	9	88	229	318	358	86	172	500	208	310	8	100	248
Fruits and Natural juices	991	816	391	762	1378	430	286	218	378	588	543	479	215	429	736	1070	858	554	843	1388	729	682	280	534	945
All vegetables	237	239	94	167	300	103	122	36	72	125	155	125	70	125	203	163	147	72	127	196	177	179	71	128	221
Leafy vegetables	73	116	17	43	81	16	20	3	9	20	46	49	14	31	60	12	21	1	4	13	48	78	8	26	59
Sweetened beverages	481	669	67	273	670	167	335	23	58	165	359	474	71	214	500	76	109	0	28	86	350	532	36	165	500
Soft drinks	229	534	0	98	206	167	335	23	58	165	‡.a	.a	.a	.a	.a	48	86	0	14	56	185	451	0	56	188
Beans	256	247	60	140	280	194	157	80	200	200	173	116	83	156	234	124	99	69	80	160	199	180	78	156	280
Fish	19	66	0	0	22	19	37	0	8	21	13	47	0	3	13	20	33	0	8	17	16	52	0	1	17
Dairy products	490	455	189	443	669	265	232	91	185	421	372	339	143	279	518	258	220	95	199	413	388	373	131	285	528
Milk	383	389	103	240	480	220	215	71	165	413	305	324	57	250	500	198	198	66	165	301	311	333	71	240	480

SD: standard deviation; * Q1: first quartile; † Q3: third quartile; ‡.a: variable not collected.

## Data Availability

The BMCNC is managed by a team of researchers from the Nutritional Epidemiology Observatory, from the Nutrition Institute, at the Federal University of Rio de Janeiro. Datasets are not yet available for public use, but requests can be made to the coordinator of the project (gilbertokac@gmail.com), and the whole consortium group is consulted regarding data sharing for specific studies.

## Supplementary Materials

The following supporting information can be downloaded at: https://www.mdpi.com/article/10.3390/nu18132068/s1, Box S1: Harmonisation of weekly food consumption categories assessed using food consumption screeners; Box S2: Description of the methodology used to assess food consumption frequency, portion sizes, and challenges encountered during the data cleaning process in each dataset derived from food frequency questionnaires; Box S3: Description of dietary intake variables obtained from food frequency questionnaires in each dataset. Figure S1: Description of the diet data harmonisation process of the Brazilian Maternal and Child Nutrition Consortium.

## List of Abbreviations

BMCNC: Brazilian Maternal and Child Nutrition Consortium, FCS: food consumption screeners, FFQ: food frequency questionnaires, BMI: body mass index, SD: standard deviations, VPC: variance partition coefficient, ICC: intraclass correlation coefficient, SISVAN: Food and Nutrition Surveillance System.

## Appendix A. Brazilian Maternal and Child Nutrition Consortium Members

Adauto Emmerich Oliveira (Postgraduate Program in Collective Health, Federal University of Espírito Santo, Vitória, ES, Brazil), Alane Cabral Menezes de Oliveira (Postgraduate Program in Nutrition and Postgraduate Program of Health Science, Institute of Biological Sciences and Health, Federal University of Alagoas, Alagoas, AL, Brazil), Ana Paula Esteves Pereira (Department of Epidemiology and Quantitative Methods in Health, Sérgio Arouca National School of Public Health, Oswaldo Cruz Foundation, Rio de Janeiro, RJ, Brazil), Ana Paula Sayuri Sato (Department of Epidemiology, School of Public Health, University of São Paulo, São Paulo, SP, Brazil), Antônio Augusto Moura da Silva (Postgraduate Program in Public Health, Federal University of Maranhão, São Luís, MA, Brazil), Carolina Abreu de Carvalho (Postgraduate Program in Public Health, Federal University of Maranhão, São Luís, MA, Brazil), Claudia Leite de Moraes (Department of Epidemiology, Institute of Social Medicine, Rio de Janeiro State University, Rio de Janeiro, RJ, Brazil), Claudia Saunders (Department of Nutrition and Dietetics, Josué de Castro Nutrition, Federal University of Rio de Janeiro, Rio de Janeiro, RJ, Brazil), Cristina Maria Garcia de Lima Parada (Nursing Department, Botucatu Medical School, Julio de Mesquita Filho Paulista State University, Botucatu, SP, Brazil), Dayana Rodrigues Farias (Nutritional Epidemiology Observatory, Department of Social and Applied Nutrition, Josue de Castro Nutrition Institute, Federal University of Rio de Janeiro, Rio de Janeiro, RJ, Brazil), Denise Cavalcante de Barros (National School of Public Health, Oswaldo Cruz Foundation, Rio de Janeiro, RJ, Brazil), Denise Petrucci Gigante (Postgraduate Program in Epidemiology, Federal University of Pelotas, Pelotas, RS, Brazil), Djanilson Barbosa dos Santos (Health Science Center, Federal University of Recôncavo da Bahia, BA, Brazil), Edson Theodoro dos Santos Neto (Postgraduate Program in Collective Health, Federal University of Espírito Santo, Vitória, ES, Brazil), Elisa Maria de Aquino Lacerda (Department of Nutrition and Dietetics, Josué de Castro Nutrition, Federal University of Rio de Janeiro, Rio de Janeiro, RJ, Brazil), Elizabeth Fujimori (Public Health Nursing Department, School of Nursing, University of São Paulo, São Paulo, SP, Brazil), Fernanda Garanhani Surita (Department of Obstetrics and Gynecology, School of Medical Sciences, University of Campinas, Campinas, SP, Brazil), Flávia Farias Lima (Federal University of Rio de Janeiro- campus Macaé, Rio de Janeiro, RJ, Brazil), Gilberto Kac (Nutritional Epidemiology Observatory, Department of Social and Applied Nutrition, Josué de Castro Nutrition Institute, Federal University of Rio de Janeiro, Rio de Janeiro, RJ, Brazil), Iracema de Mattos Paranhos Calderon (Department of Gynecology and Obstetrics, Botucatu School of Medicine, Paulista State University Júlio de Mesquita Filho), Isabel Oliveira Bierhals (Postgraduate Program in Epidemiology, Federal University of Pelotas, Pelotas, RS, Brazil), Isaac Suzart Gomes-Filho (Department of Health, Feira de Santana State University, Feira de Santana, BA, Brazil), Jane de Carlos Santana Capelli (Federal University of Rio de Janeiro, campus Macaé, Macaé, RJ, Brazil), Jerusa da Mota Santana (Health Science Center, Federal University of Recôncavo da Bahia, BA, Brazil), José Guilherme Cecatti (Department of Obstetrics and Gynecology, School of Medical Sciences, University of Campinas, Campinas, SP, Brazil), Juraci Almeida Cesar (Postgraduate Program in Public Health, Federal University of Rio Grande, Rio Grande, RS, Brazil), Katrini Guidolini Martinelli (Post-Graduate Program in Collective Health, Federal University of Espírito Santo), Lívia Castro Crivellenti (Postgraduate Program in Public Health, Department of Social Medicine, Faculty of Medicine of Ribeirão Preto, University of São Paulo), Luana Patrícia Marmitt (Post-Graduate Program in Bioscience and Health, University of Western Santa Catarina), Maria Angélica A Nunes (Postgraduate Studies Program in Epidemiology, School of Medicine, Federal University of Rio Grande do Sul), Maria Antonieta de Barros Leite Carvalhaes (Julio de Mesquita Filho Paulista State University, Botucatu, SP, Brazil), Maria do Carmo Leal (Department of Epidemiology and Quantitative Methods in Health, National School of Public Health, Oswaldo Cruz Foundation), Maria Inês Schmidt (Postgraduate Studies Program in Epidemiology, School of Medicine, Federal University of Rio Grande do Sul, RS, Brazil), Maria Fernanda Larcher de Almeida (Federal University of Rio de Janeiro, campus Macaé), Mayra Pacheco Fernandes (Postgraduate Program in Epidemiology, Federal University of Pelotas, Pelotas, RS, Brazil), Michael Eduardo Reichenheim (Department of Epidemiology, Institute of Social Medicine, Rio de Janeiro State University), Nathalia Cristina de Freitas-Costa (Nutritional Epidemiology Observatory, Department of Social and Applied Nutrition, Josue de Castro Nutrition Institute, Federal University of Rio de Janeiro), Patrícia de Carvalho Padilha (Department of Nutrition and Dietetics, Josué de Castro Nutrition, Federal University of Rio de Janeiro, Rio de Janeiro, RJ, Brazil), Renato Passini Junior (Department of Obstetrics and Gynecology, School of Medicine, University of Campinas, Campinas, SP, Brazil), Renato Teixeira Souza (Department of Obstetrics and Gynecology, School of Medical Sciences, University of Campinas, Campinas, SP, Brazil), Silvia Regina Dias Medici Saldiva (Department of Pathology, Medical School of the University of São Paulo, São Paulo, SP, Brazil), Silvio O. M. Prietsch (Postgraduate Program in Public Health, Federal University of Rio Grande, Rio Grande, RS, Brazil), Simone Seixas da Cruz (Department of Epidemiology, Federal University of Recôncavo da Bahia, Santo Antônio de Jesus, BA, Brazil), Sirlei Siani Morais (Department of Obstetrics and Gynecology, School of Medical Sciences, University of Campinas, Campinas, SP, Brazil), Sotero Serrate Mengue (Postgraduate Program in Epidemiology, Department of Social Medicine, School of Medicine, Federal University of Rio Grande do Sul, Porto Alegre, RS, Brazil), Thaís Rangel Bousquet Carrilho (Nutritional Epidemiology Observatory, Department of Social and Applied Nutrition, Josue de Castro Nutrition Institute, Federal University of Rio de Janeiro), Vera Lúcia Bosa (Post-Graduate Program in Food, Nutrition and Health, Department of Nutrition, Federal University of Rio Grande do Sul).
