# Supplementary material for: Harmonisation of Dietary Intake Data in Pregnant Women: Data from the Brazilian Maternal and Child Nutrition Consortium—BMCNC
Source: Nutrients. 2026 Jun 24;18(13):2068. doi: 10.3390/nu18132068 (PMC13362956; doi:10.3390/nu18132068)
Supplement: Supplementary file 1 [file nutrients-18-02068-s001.zip › Figure Suplementary 1.pdf]

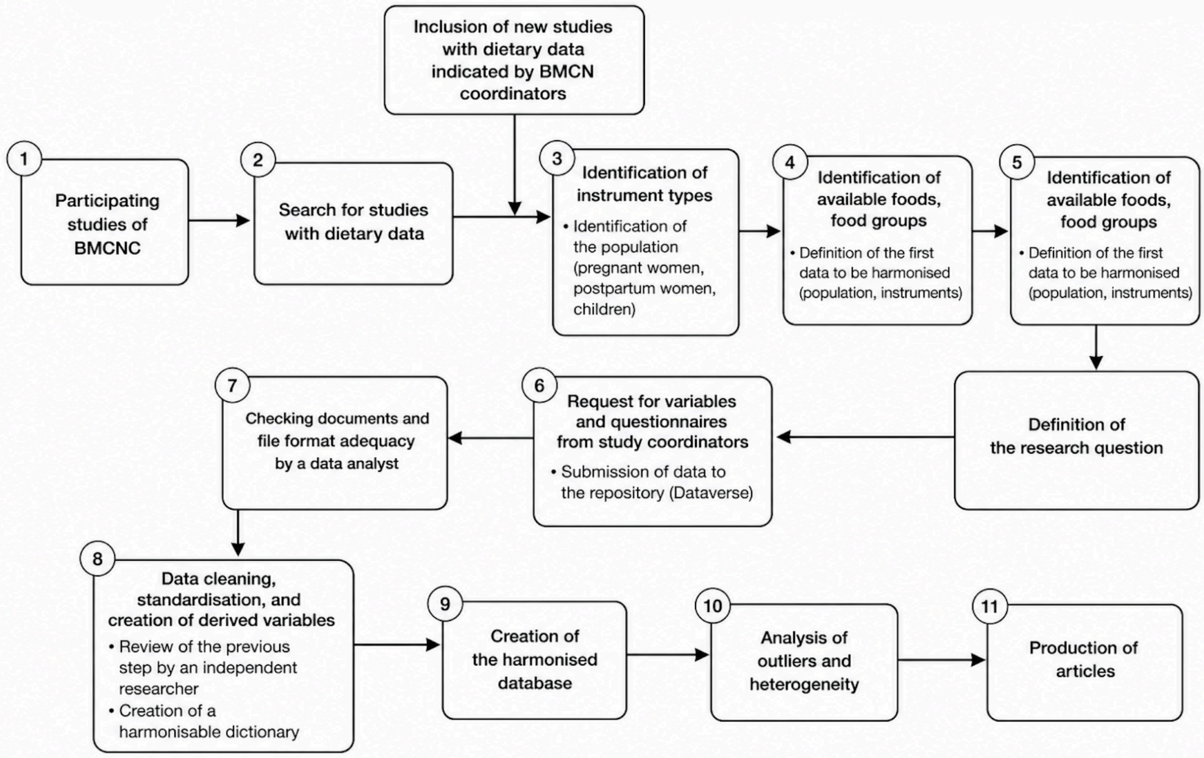

Figure S1. Description of the diet data harmonisation process of the Brazilian Maternal and Child Nutrition Consortium.
