# Supplementary material for: Harmonisation of Dietary Intake Data in Pregnant Women: Data from the Brazilian Maternal and Child Nutrition Consortium—BMCNC
Source: Nutrients. 2026 Jun 24;18(13):2068. doi: 10.3390/nu18132068 (PMC13362956; doi:10.3390/nu18132068)
Supplement: Supplementary file 1 [file nutrients-18-02068-s001.zip › nutrients-4266925-supplementary (1).pdf]

Supplementary Material

Box S1. Harmonisation of weekly food consumption categories measured by food consumption screeners.

Original Category	[a]Carvalhaes *et al.*	[b]Gomes *et al.*	[c]Silva *et al.*	[d]Ferreira *et al.*	[e]Marano D *et al.*	[f]Santos *et al.*	[g]Polgliani *et al.*	[h]Martinelli *et al.*	Harmonised data
foods/food groups belonging to each database	fruits, vegetables, soft drinks and milk.	fruits, natural juice, vegetables, leafy vegetables, soft drinks, artificial juice, beans and milk.	milk	fruits, natural juice, vegetables, soft drinks, beans and milk.	fruits and natural juice, vegetables and milk.	fruits, natural juice, vegetables, soft drinks, artificial juice, beans and milk.	fruits, natural juice, vegetables, soft drinks, artificial juice, beans and milk.	fruits, natural juice, vegetables, soft drinks, artificial juice, beans and milk.	All items
Daily	x	x		x	x	x	x	x	≥ 5 days or more per week
≥5 times/week*			x						
5-6 days/week	x	x				x	x	x	
3-4 days/week	x	x				x	x	x	1-4 days per week
>3 times/week					x				
3-4 times/week			x						
2-3 times/week				x					
1-3 times/week					x				
1-2 days/week	x	x				x	x	x	
1-2 times/week			x						
1 times/week				x					
2-3 times/month			x						Never/ almost never
≤1 times/month			x						
Almost never	x	x		x	x	x	x	x	
Never	x	x		x	x	x	x	x	

[a]Pregnancy cohort study carried out in Botucatu, SP (Carvalhaes MA *et al.*, 2013); [b]Pregnancy cohort study carried out in Botucatu, SP(Gomes CB *et al.*, 2020); [c]Coorte de pré-natal brasileira de Ribeirão Preto e São Luís (Silva AA *et al.*, 2014); [d]Nutritional status, weight gain and feeding behaviour of pregnant women in Maceió-Alagoas: impact on health of the binomial mother-child (Ferreira RC *et al.*, 2020); [e]Study on Social Capital and Psychosocial Factors Associated with Prematurity and Low Birth Weight - Cohort in two municipalities of Rio de Janeiro (Marano *et al.*, 2012); [f]Study on SUS assistance in Greater Vitória, ES (Santos ET *et al.*, 2012); [g]Assessment of the Quality of Prenatal Care in the Metropolitan Region of Greater Vitória, ES (Polgliani *et al.*, 2014); [h]Assessment of Prenatal Care in the São Mateus Microregion, ES (Martinelli KG *et al.*, 2014); *Not daily.

Box S2 - Description of the methodology used to assess food consumption frequency, portion sizes, and challenges encountered during the data cleaning process in each dataset derived from food frequency questionnaires.

Dataset	Method used to assess consumption frequency	Consumption frequency categories	Method used to assess portion size	Reference used for portion size estimation in the FFQs	Foods without portion size
[a]**Nunes *et al*.**	For each item in the questionnaire, consumption frequency was measured using 8 response options, ranging from daily, weekly, or monthly consumption.	>3 times/day; 2 to 3 times/day; 1 time/day; 5 to 6 times/week; 2 to 4 times/day; 1 time/week; 1 to 3 times/month; never/almost never.	Respondents were given a choice of household measures for each item.<br><br>Standardised household measure (numeric variable with 3 digits and 1 decimal place - ##.#)	Pinheiro ABV, Lacerda EMA, Benzecky EH, Gomes MCS, Costa VM. Table for evaluating food consumption in household measures. 5th ed. São Paulo: Atheneu Publisher; 2005.	For 10 items, only consumption frequency information was collected: garlic, onion, bell pepper, lemon, passion fruit, butter, margarine, cream cheese, candies/caramels, and other alcoholic beverages.
[b]**Farias *et al.***	For each item in the questionnaire, consumption frequency was measured using 8 response options, including daily or weekly consumption.	>3 times/day; 2 to 3 times/day; 1 time/day; 5 to 6 times/week; 2 to 4 times/week; 1 time/week.	The respondent was asked to choose one category from among 2-3 size categories presented.	Pinheiro ABV, Lacerda EMA, Benzecky EH, Gomes MCS, Costa VM. Table for evaluating food consumption in household measures. 5th ed. São Paulo: Atheneu Publisher; 2005.	For 19 items, only consumption frequency information was collected: garlic, onion, bell pepper, lemon, passion fruit, butter, margarine, cream cheese, candies, other alcoholic beverages, peanuts (snack), bacon/crackling/pork fat, lard, tripe/gizzard/liver/heart, jerked meat/cod, barbecue, lasagna, mortadella/ham, popcorn, chips, canned tuna/sardines, and other canned foods.

Dataset	Method used to assess consumption frequency	Consumption frequency categories	Method used to assess portion size	Reference used for portion size estimation in the FFQs	Foods without portion size
[c]**Sartorelli *et al.***	For each item in the questionnaire, consumption frequency was measured with 4 response options: daily, weekly, monthly, or annually. It also measured the number of times the item was consumed per occasion, ranging from 1 to 10 times.	Daily, weekly, monthly, annually<br><br>How many times? 1,2,3,4,5,6,7,8,9,10	For each item, portion size was estimated using an average reference value, allowing the respondent to choose the portion size consumed from 4 options (small, medium, large, extra large).	Based on 24-hour dietary recalls applied to the same population, the 25th, 50th, 75th, and 100th percentiles were used to determine the sizes for small, medium, large, and extra-large portions, respectively.<br><br>Oliveira, T., Marquitti, F.D., Carvalhaes, M.A. & Sartorelli, D.S. (2010). Development of a Quantitative Food Frequency Questionnaire (FFQ) for pregnant women attending primary health care units in Ribeirão Preto, São Paulo, Brazil. Cad. Saúde Pública 26, 2296–2306.	---
[d]**Santana *et al.***	For each item in the questionnaire, consumption frequency was measured with 8 response options, including daily, weekly, or monthly consumption.	> 3 times/day; 2 to 3 times/day; 1 time/day; 5 to 6 times/week; 2 to 4 times/week; 1 time/week; 1 to 3 times/month; never/almost never	The respondent was given a choice of household measure for each item.	MONTEIRO, J. P. Food Consumption Visualizing Portions. Guanabara Koogan; 2007.	For 9 items, only consumption frequency information was collected: garlic, onion, bell pepper, lemon, passion fruit, butter, margarine, cream cheese, and candies/caramels.

[b]Study of Consumption and Eating Behavior in Pregnant Women (Nunes *et al.*, 2010); [c]Cohort Study during Pregnancy in Rio de Janeiro (Farias *et al.*, 2014); [d]GDM Study in Ribeirão Preto (Sartorelli DS *et al.*, 2019); [f] Longitudinal study in a region of the north east of Brazil (Santana J *et al*., 2015).

Box S3. Description of dietary intake variables from food frequency questionnaires in each dataset.

**Food group**	**Studies**			
	[a]**Nunes *et al.***	[b]**Farias *et al.***	[c]**Sartorelli *et al.***	[d]**Santana *et al.***
**Fruits**	13 items (orange/bergamot, banana, papaya, apple, watermelon/melon, pineapple, avocado, mango, lemon*, passion fruit*, grape, guava, pear)	8 items (orange/tangerine, banana, papaya, apple, watermelon/melon, pineapple, mango, grape)	12 items (pineapple, banana, bergamot/orange, guava, mango/persimmon, apple/pear, watermelon/melon, papaya/papaya Formosa, strawberry, peach, avocado/blended avocado pulp, grape)	14 items (orange bergamot, banana, papaya, apple, watermelon/melon, pineapple, avocado, mango, lemon*, passion fruit*, guava, plantain, tangerine, breadfruit)
**Natural juices**	1 item (natural fruit juice)	1 item (fruit juice or pulp juice)	2 items (orange juice, other natural fruit juice)	1 item (natural juice)
**Fruits and e natural juices**	14 items (orange, banana, papaya, apple, watermelon, pineapple, avocado, mango, lemon, passion fruit, grape, guava, pear, natural fruit juice)	9 items (orange, banana, papaya, apple, watermelon, pineapple, mango, grape, fruit or pulp juice)	14 items (pineapple, banana, tangerine/orange, guava, mango/persimmon, apple/pear, watermelon/melon, papaya/papaya Formosa, strawberry, peach, avocado/blended avocado pulp, grape, orange juice, other natural fruit juice)	15 items (orange/tangerine, banana, papaya, apple, watermelon/melon, pineapple, avocado, mango, lemon*, passion fruit*, guava, plantain, tangerine, breadfruit, jackfruit, natural fruit juice)
**Leafy vegetables**	4 items (lettuce, collard greens, cabbage, chicory)	3 items (lettuce, collard greens, cabbage)	2 items (lettuce/chinese cabbage/ cabbage, watercress/lettuce/ rocket/collard greens)	3 items (lettuce, collard greens, cabbage)
**Vegetables**	10 items (bell pepper, carrot, beetroot, cauliflower, tomato, chayote, pumpkin, courgette, cucumber, green beans)	11 items (cauliflower/ broccoli, tomato, raw cucumber, chayote, courgette, pumpkin, carrot, beetroot, okra, green beans, bell pepper*)	8 items (beetroot, carrot, cucumber, tomato, pumpkin, courgette, broccoli, and another vegetable such as green beans, chayote, or cauliflower)	7 items (tomato, chayote, pumpkin, cucumber, bell pepper*, carrot, and scarlet eggplant)
**All vegetables**	14 items (lettuce, collard greens, cabbage, chicory, bell pepper*, carrot, beetroot, cauliflower, tomato, chayote, pumpkin, courgette, cucumber, green beans)	14 items (lettuce, collard greens, cabbage, cauliflower, tomato, cucumber, chayote, courgette, pumpkin, carrot, beetroot, okra, green beans, bell pepper*)	10 items (lettuce/chinese cabbage/ cabbage, watercress/endive/ rocket/collard greens, beetroot, carrot, cucumber, tomato, pumpkin, courgette, broccoli, other vegetable such as: green beans, chayote, or cauliflower)	10 items (lettuce, collard greens, cabbage, tomato, chayote, pumpkin, cucumber, bell pepper*, carrot, scarlet eggplant)
**Soft drinks**	2 items (soft drink, light soft drink)	2 items (cola-based soft drink, Other soft drinks , guaraná ou Guaravita)	-	1 item (soft drink)
**Sweetened beverages**	3 items (soft drink, light soft drink, artificial juice)	2 items (cola-based soft drink, others soft drinks, guaraná or Guaravita)	1 item (artificial juice or soft drink)	2 items (soft drink, artificial juice)
**Beans**	1 item (beans)	1 item (beans)	2 items (cooked beans, seasoned beans)	1 item (beans)

**Food group**	**Studies**			
	[a]**Nunes *et al.***	[b]**Farias *et al.***	[c]**Sartorelli *et al.***	[d]**Santana *et al.***
**Fish**	1 item (fresh fish)	1 item (fresh fish)	2 items (cooked fish, fried fish)	1 item (fresh fish)
**Milk**	3 items (whole milk, semi-skimmed milk, skimmed milk)	1 item (milk with an option to specify whether it is whole, semi-skimmed, or skimmed)	2 items (whole milk, skimmed milk)	1 item (milk with an option to specify whether it is whole, semi-skimmed, or skimmed)
**Dairy products**	6 items (whole milk, semi-skimmed milk, skimmed milk, yoghourt, light yoghourt, cheese)	3 items (milk with an option to specify whether it is whole full-fat, semi-skimmed, or skimmed, yoghourt, cheese)	6 items (whole milk, skimmed milk, full-fat yoghurt, skimmed yoghourt, yellow cheese, white cheese)	3 items (whole milk or semi-skimmed milk or skimmed milk, regular yoghourt or light/skimmed yoghourt, cheese)

[a]Study of Consumption and Eating Behavior in Pregnant Women (Nunes *et al*., 2010); [b]Cohort Study during Pregnancy in Rio de Janeiro, RJ (Farias *et al*., 2014); [c]GDM Study in Ribeirão Preto (Sartorelli DS *et al*., 2019); [d]Longitudinal study in a region of the north east of Brazil (Santana J *et al*., 2015). *Foods without quantity information—only frequency of consumption.
